# Outcomes in acute kidney injury in noncritically ill patients lately referred to nephrologist in a developing country: a comparison of AKIN and KDIGO criteria

**DOI:** 10.1186/s12882-020-01751-7

**Published:** 2020-03-11

**Authors:** Ginivaldo Victor Ribeiro do Nascimento, Marcela do Nascimento Silva, Juarez Duarte de Carvalho Neto, Ludgero Ribeiro Feitosa Filho, Jessica Duarte Antão

**Affiliations:** 1 Centro de Ciências da Saúde – FACIME/UESPI – Coordenação de Medicina, Universidade Estadual do Piauí, Rua Olavo Bilac, 2335, Centro, Teresina, PI CEP 64001-280 Brazil; 2 FACID/Wyden - Coordenação Medicina, Teresina, Brazil; 3 Uninovafapi - Coordenação Medicina, Teresina, Brazil

**Keywords:** Acute kidney injury, Nephrologist referral, Nephrologist consultation, Developing country, Dialysis

## Abstract

**Background:**

In low-middle-income countries (LMICs), data regarding acute kidney injury (AKI) are scarce. AKI patients experience delayed diagnosis. This study aimed to evaluate whether delayed nephrologist consultation (NC) affected outcomes of AKI patients and compare Acute Kidney Injury Network (AKIN) and Kidney Disease: Improving Global Outcomes (KDIGO).

**Methods:**

An observational, retrospective study was conducted in a tertiary public hospital in an LMIC.

**Results:**

Overall, 103 AKI patients were analysed. In-hospital mortality was 61.16%, and dialysis was required in 38.83%. NC took place after 48 h in 68.93% of the patients. Mean time for NC was 5.22 ± 4.30 days. At NC, serum creatinine was 4.48 (±3.40) mg/dL and blood urea nitrogen was 68.21 (± 35.02) mg/dL. The AKIN and KDIGO stage stratifications were identical; KDIGO stage 3 was seen in 58.25% of the patients. The group with NC >  4 days had a mortality rate of 74.46% and the group with NC ≤ 4 days had a mortality rate of 50% (*p* = 0.011). Multivariate analysis showed that haemodialysis was independently associated with mortality. NC >  4 days was associated with death [odds ratio 2.66 (95% confidence interval, 1.36–4.35), *p* = 0.001]. Logistic regression showed an OR of 1.20 (95% CI, 1.05–1.37) (*p* = 0.008) for each day of delayed NC.

**Conclusion:**

Delayed NC was associated with mortality even after adjustments, as was haemodialysis, though marginally. In AKI patients with NC > 4 days, there was a high prevalence of KDIGO stage 3, and AKIN and KDIGO criteria were identical.

## Background

Despite technological and conceptual improvements in acute kidney injury (AKI) assistance, mortality has declined only slowly. Furthermore, the need for dialysis reaches 80% of patients in some intensive care units (ICU) [1–6]. Conversely, a recent report of global epidemiology shows that only 11% of all AKI cases require dialysis in hospital facilities [3].

The general trend toward an increasing severity of illness in ICU patients and AKI presenting not in isolation but usually as a complication of several diseases could partly explain this scenario [5, 7, 8]. Another reason is that delayed nephrology consultations prevent timely interventions that provide opportunities for the modification of AKI patient outcomes [9, 10].

The term acute kidney injury has now emphasized a continuum of kidney injury, with an important contribution made by the introduction of Risk, Injury, Failure, Loss, and End-Stage Kidney Disease (RIFLE) and Acute Kidney Injury Network (AKIN) criteria [11, 12], a system for the diagnosis and classification of a broad range of acute kidney function impairments. A new consensus definition has emerged from the Kidney Disease: Improving Global Outcomes (KDIGO) group who intended to harmonize definitions and staging systems from prior guidelines. Their definition has been validated in thousands of patients and appears to perform better than AKIN and RIFLE [13]. However, the adjustments for confounding factors did not include the time for nephrology consultation in these series.

More than 85% of the world’s population resides in low-income and middle-income countries, where there is commonly a paucity of data regarding AKI [3]. Socioeconomic and environmental factors such as tropical febrile illnesses, envenoming, and obstetrical complications influence the epidemiology of AKI [3, 14–17]. In metropolitan regions, the clinical profile of AKI patients may be very similar to that encountered in developed countries [3]. Additionally, availability of trained personnel and access to diagnostic tests and dialysis affect practice patterns and impose barriers to care [3, 14–17]. The extent to which these factors contribute to mortality and non-recovery of renal function has not been quantified [3].

Some reports have indicated that early nephrology consultation may improve critical and noncritical AKI prognosis, although none of these studies have compared the established sets of criteria for the diagnosis of AKI (AKIN and KDIGO criteria) and examined prediction of in-hospital mortality [9, 18, 19].

The aims of this study were to evaluate the outcomes of a population of AKI patients who had lately been referred to a nephrologist and to determine whether KDIGO could be superior to AKIN in this setting.

## Methods

Study participants: A retrospective, observational study was conducted through a search for AKI cases referred to the nephrology team at Hospital de Urgências de Teresina, a tertiary public hospital urgency centre in Brazil.

This study was reviewed and approved by the local Committee of Research Ethics who waived the need for written informed consent from the participants of the study.

All patients consecutively admitted to the hospital and presenting with AKI assisted by the nephrology team were evaluated between January 2011 and December 2011. All AKI patients were reviewed from the day of nephrology consultation until recovery of renal function, hospital discharge, or death.

Only two researchers, who were not involved in patient care, collected data. The day of nephrology referral was considered the day of the nephrologists’ call, because the attending nephrologists are available 24 h each day and the consultation was usually performed immediately after the call on the same day. The next step was to search at the data for the time of AKI.

AKI was defined as an increase of > 0.3 mg/dL from the baseline serum creatinine (SCr) within 48 h or an increase in SCr to 1.5 times baseline, which is known or presumed to have occurred within 7 days, according to the AKIN and KDIGO criteria, respectively. Patients with a SCr of 1.5 mg/dL or more, without known baseline SCr values and without SCr decrease, were viewed as having AKI only if history, renal ultrasound, and laboratory examinations were indicative of this diagnosis [20]. In this case, we used an estimated baseline SCr or the lowest SCr value during their stay in the hospital, whichever was lower. The baseline SCr was estimated using the simplified Modification of Diet in Renal Disease (MDRD) formula, assuming a glomerular filtration rate (GFR) of 75 mL/min per 1.73 m^2^ [21, 22]. The exclusion criteria were: patients on chronic dialysis treatment; age < 18 years old; kidney transplantation; patients without known previous SCr, whose SCr did not normalize (≤ 1.5 mg/dL), or whose SCr did not decrease by at least 50% from its peak value during hospitalization; patients without data concerning the time of nephrologist referral; and patients with glomerulopathy AKI, similarly to that used by Santos et al. [20]

Each patient’s chart was reviewed after identifying the day of AKI diagnosis, and a cause of kidney injury was determined based on available clinical and laboratory data.

Decreased renal perfusion was identified by observations of signs of volume depletion on physical examination, a decrease in blood pressure, clinical evidence of congestive heart failure, improvement with restoration of renal blood flow, and the absence of other causes of kidney injury [20, 23].

Contrast-induced nephropathy was defined as an AKI cause when SCr level increased within 48 h after intravenous contrast administration [23].

Nephrotoxic drugs were defined as an AKI cause when the increase in SCr level was temporally related to administration of the medication, based on clinical or laboratory evidence supporting acute tubular necrosis, interstitial nephritis, or hemodynamic effect [23].

Post-renal AKI was considered the cause of kidney damage if there was evidence of obstruction on radiographic studies and improvement in renal function with relief of obstruction [23].

The following variables were collected: age, sex, race, hospital admission days, co-morbidities, baseline renal function, presumed AKI etiologies, urine output, and laboratory tests, similarly to that used by Costa e Silva [10] The primary outcome was in-hospital mortality. Nephrologist referral, indication of dialysis, and clinical and laboratory characteristics were also recorded.

Statistical analysis: Continuous variables were expressed as mean ± SD or median with 25th and 75th interquartile ranges (IQR) according to the normality of their distribution using the Kolmogorov-Smirnov test. Categorical variables were expressed as proportions and were compared with Pearson’s x [2] test. Multivariable logistic regression models was performed using backwards variable selection, using *P*-value < 0.05 for variable retention. Candidate variables were those with a likelihood ratio of significance < 0.2 upon bivariate analysis [2, 10, 24, 25]. Variables were checked for multicollinearity performing Variance Inflation Factor. However, multicollinearity was not detected.

All tests of significance were two-sided, with a *p*-value of < 0.05 indicating statistical significance. The data were analyzed using the Statistical Package for the Social Sciences version 20.0 (SPSS, Chicago, IL, USA).

## Results

**Fig. 1:**
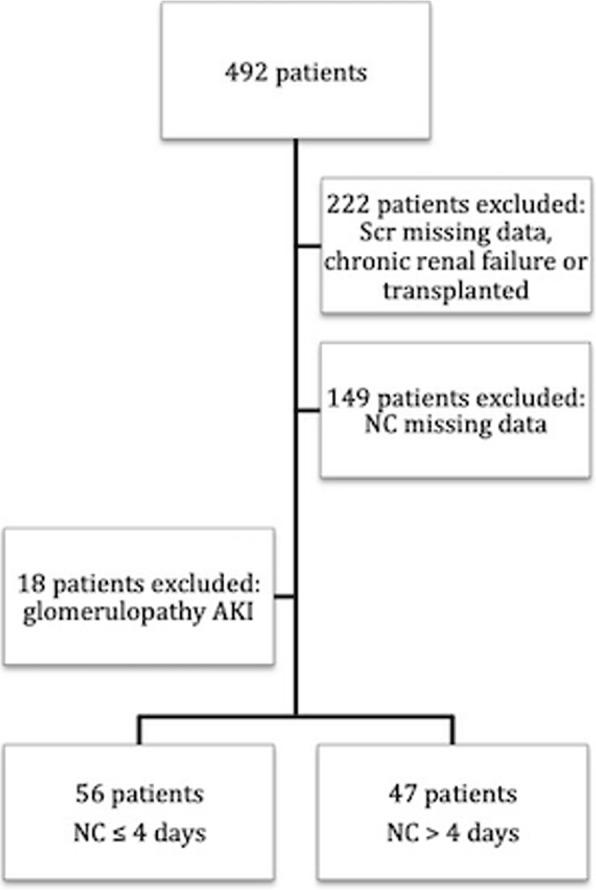
Flowchart of study Outcomes AKI. AKI: acute kidney injury; Scr: serum creatinine; NC: Nephrologist consultation

The nephrologist team followed a total of 492 patients during the study period. Of these, 222 were excluded according to the study criteria, with 270 AKI patients remaining; 149 of these were excluded for lacking accurate data regarding time of nephrologist consultation and 18 were excluded for glomerulopathy AKI. Thus, a total of 103 AKI patients were included for analysis (Fig. 1). Delayed NC occurred in 68.93% of patients; the mean time was 5.22 ± 4.3 days after the day of AKI diagnosis. The overall in-hospital mortality rate was 61.16%, and dialysis was required in 38.83% of patients.

Clinical and laboratory characteristics are shown in Table 1. Of the patients, 67.96% (70) were male, 61.16% (63) were older than 60 years old, and 70.87% (73) were oliguric. Ischemia was the most prevalent aetiology at 45.63%. SCr at NC was 4.48 mg/dL (± 3.40 mg/dL), and blood urea nitrogen (BUN) at NC was 68.21 mg/dL (± 35.02 mg/dL). Fifty eight percent of patients (60) were stratified at AKIN and KDIGO stage 3, followed by 34% classified as stage 1. Only 6 patients were at stage 2 of AKIN and KDIGO. Forty-seven patients (45.63%) waited more than 4 days for a nephrologist consultation after they met criteria for AKI.

**Table 1:** Baseline clinical and laboratory characteristics of AKI patients lately referred to nephrologist in developing country (*n* = 103)

Characteristics	n = 103
**Male**	70 (67.96)
**Age ≥ 60 years**	63 (61.16)
**Oliguria**	73 (70.87)
**Etiology**	
Ischemic	47 (45.63)
Sepsis	21 (20.38)
Multifatorial	18 (17.43)
**Serum creatinine** (mg/dL)	
Baseline	4.48 (3.40)
Maximun	5.37 (3.15)
**Blood urea nitrogen** (mg/dL)	
Baseline	68.21 (35.02)
Maximum	93.83 (34.81)
**Serum potassium** (mg/dL)	
Baseline	4.64 (1.24)
Maximum	5.09 (1.12)
**AKIN**	
stage I	35 (33.98)
stageII	6 (5.82)
stage III	60 (58.25)
**KDIGO**	
stage I	35 (33.98)
stage II	6 (5.82)
stage III	60 (58.25)
**Nephrologist consultation**	
≤ 4 days	56 (54.36)
> 4 days	47 (45.63)
**Hemodialysis**	40 (38.83)
**Mortality**	63 (61.16)

Results are expressed in number (%), mean ± SD or median (25–75 IQR). AKIN: Acute Kidney Injury Network. KDIGO: Kidney Disease: Improving Global Outcomes

Table 2 shows the univariate analysis of the clinical and lab variables associated with death. Gender, age > 60 years, oliguria, baseline BUN, AKIN, and KDIGO, were not associated with death. Only NC > 4 days, baseline SCr, maximum Scr, and haemodialysis were associated with mortality.

**Table 2:** Association between baseline clinical and laboratory characteristics and nephrology consultation of AKI patients lately referred to nephrologist in developing country (*n* = 103)

Characteristics	Nephrology consultation		*p*
	≤ 4 days (*n* = 56)	> 4 days (*n* = 47)	
**Male**	38 (67.85)	40 (85.11)	0.980
**Age ≥ 60 years**	37 (66.07)	26 (55.32)	0.265
**Oliguria**	38 (67.85)	35 (74.47)	0.462
**Serum creatinine** (mg/dL)			
Baseline	3.1 (2.47)	4.1 (4.11)	0.198
Maximum	4.5 (2.92)	4.6 (3.38)	0.292
**Blood urea nitrogen** (mg/dL)			
Baseline	66.57 (31.58)	70.17 (38.99)	0.606
Maximum	94 (37.96)	93.63 (31.3)	0.959
**Serum potassium** (mg/dL)			
Baseline	4.61 (1.07)	4.69 (1.43)	0.758
Maximum	5.14 (1.22)	5.05 (1.01)	0.675
**AKIN**			
stage I	21 (38.88)	14 (29.78)	0.437
stage II	4 (7.41)	2 (4.25)	
stage III	29 (53.7)	31 (65.95)	
**KDIGO**			
stage I	21 (38.88)	14 (29.78)	0.437
stage II	4 (7.41)	2 (4.25)	
stage III	29 (53.7)	31 (65.95)	
**Hemodialysis**	38 (67.85)	47 (68.11)	0.128
**Mortality**	28 (50.0)	35 (74.46)	0.011

Results are expressed in number (%), mean ± SD or median (25–75 IQR). AKIN: Acute Kidney Injury Network. KDIGO: Kidney Disease: Improving Global Outcomes

There were no baseline differences between early and delayed consultation groups (Table 3). The delayed NC group was associated with mortality 74.46% vs 50% (*p* = 0.011). Multivariate analysis showed that baseline and maximum SCr and haemodialysis were independently associated with mortality (Table 4). NC > 4 days was associated with death [odds ratio (OR) 2.66 (95% confidence interval – CI: 1.36–4.35), *p* = 0.001]. Logistic regression showed an OR of 1.20 (95% CI, 1.05–1.37); *p* = 0.008, for each day of delayed consultation.

**Table 3:** Association between baseline clinical and laboratory characteristics and mortality of AKI patients lately referred to nephrologist in developing country (n = 103)

Variables	Survivors (*n* = 40)	Non-survivors (*n* = 63)	p
**Gender**			
Male	30 (75)	40 (63.49)	0.222
Female	10 (25)	23 (36.51)	
**Age ≥ 60 years**	25 (39.68)	38 (60.31)	0.824
**Oliguria**	28 (38.35)	45 (61.64)	0.876
**Serum creatinine** (mg/dL)			
Baseline	3 (3.02)	4 (3.57)	0.071
Maximum	4.05 (2.91)	5 (3.19)	0.011
**Blood urea nitrogen** (mg/dL)			
Baseline	59.12 (30.31)	73.99 (36.78)	0.035
Maximum	87.65 (35.66)	97.79 (33.96)	0.157
**Serum potassium** (mg/dL)			
Baseline	4.50 (1.07)	4.74 (1.34)	0.337
Maximum	5.10 (1.10)	5.10 (1.15)	0.996
**AKIN**			
stage I	18 (45.00)	17 (27.86)	0.142
stage II	3 (7.5)	3 (4.91)	
stage III	19 (47.5)	41 (67.21)	
**KDIGO**			
stage I	18 (45.0)	17 (27.86)	0.142
stage II	3 (7.5)	3 (4.91)	
stage III	19 (47.5)	41 (67.21)	
**Nephrologist consultation**			
≤ 4 days	28 (70.00)	28 (44.44)	0.011
> 4 days	12 (30.00)	35 (55.55)	
**Hemodialysis**			
Yes	30 (75.00)	33 (52.8)	0.021
No	10 (25.00)	30 (47.61)	

Results are expressed in number (%), mean ± SD or median (25–75 IQR). AKIN: Acute Kidney Injury Network. KDIGO: Kidney Disease: Improving Global Outcomes

**Table 4:** Multivariate analysis of death-related variables in AKI patients lately referred to nephrologist in developing country

Variable	OR ajusted (95% confidence interval)	P
Serum creatinine		
Baseline	1.36 (1.11–1.57)	**0.049**
Maximum	1.30 (1.02–1.71)	**0.039**
Hemodialysis	1.38 (1.01–2.53)	**0.043**
Nephrologist consultation > 4 days	2.66 (1.36–4.35)	**0.001**

OR: odds ratio

## Discussion

This report was conducted in a tertiary hospital of a low-middle-income state in Brazil (22st place of 27 states in Brazil regarding national gross domestic product [26]), the only public urgency centre from the national health insurance program for a coverage population of approximately 1 million people. A high mortality of 61.16% was observed, similar to that found in other populations [2, 3, 14, 16], principally when there is a predominance of KDIGO stage 3, as observed in this report (58.25% of all patients) [3]. Additionally, as shown by Mehta et al., 42% of patients at this stage of KDIGO require dialysis [3]. We found that 38.83% underwent renal replacement therapy.

Although a nephrologist was available at the tertiary hospital 24 h a day, NC occurred after a mean time of 5.22 ± 4.3 days of AKI diagnosis (diagnosis was confirmed after checking patients’ medical charts) and 68.9% of patients had a delayed consultation of more than 48 h. Delayed nephrologist referral may be due to unrecognized diagnosis, misunderstanding of the significance of timely intervention, and unfamiliarity with early recognition and early treatment by the attending physician [3]. Mehta et al. [24] found in a pioneering study evaluating the impact of referral to a nephrologist on mortality a NC median time of 4 days in ICU patients. Ponce et al. [9] and Costa e Silva et al. [10] also evaluated ICU patients and reported a delayed consultation in 62.33 and 34.70% of patients, respectively. In these studies, the mean time for NC were 4.7 and 3 days, respectively; less than observed in our research. Soares et al. [27] in a recent meta-analysis reported that delayed nephrology consultation is usually associated with severe stages of AKI urgent indications of renal replacement therapy, higher mortality, reduced renal recovery, higher dependency of dialysis, and higher costs.

This study was performed in a developing country, in particular a very poor state, albeit in a tertiary hospital. It shows, in agreement with Mehta et al. [3], that the AKI epidemiology (Table 1) was similar to that in developed countries and distinct from that in general LMICs. In the present study, ischemia was the major cause of AKI, unlike the multinational survey of Lombardi et al. [17] which demonstrated common low-income AKI causes. In our report, we found that 61.16% of patients were older than 60 years, similar to other studies [2, 5, 8, 9, 18, 19]. In a high-income country study assessing 1020 patients, Wonnacott et al. [28] found that patients had a mean age of 75 years. Holmes et al. [29] reported that, in low-income regions, age is an independent risk factor, particularly in the elderly. In the present study, age (> 60 years) was not associated with mortality. We found that 70.87% of patients were oliguric; this value was higher than those observed in other studies (Macedo et al. [30], 47%; Nascimento et al. [2], 37.21%; and Costa e Silva et al. [10], 25,13%), possibly reflecting the severity and late call of the nephrology team. In all patients, we found very high levels of SCr and BUN, similar to those reported by Metha et al. [24] and Ponce et al. [9]

In this population, AKIN and KDIGO criteria resulted in exactly the same classification of the patients to different AKI stages, as observed in the FINNAKI study [31]. That study, performed in an ICU, found mainly stage 1 and 3 AKI (43.73 and 35.93% respectively). We found a higher incidence of stage 3 AKI (58.25%) followed by stage 1 (33.98%). Low-middle-income countries present similar patterns of KDIGO stages, as demonstrated by Mehta et al. [15] in a multinational cross-over study, of high stage 3 incidence (58%) followed by stage 1 (29%). In a study enrolling patients hospitalized for acute decompensated heart failure, the incidence of AKI as defined by RIFLE, AKIN, and KDIGO criteria was also similar [32]. Fujii et al. [33], in a large database of 47,518 patients, found that KDIGO and RIFLE were superior to AKIN in diagnosing AKI.

It is likely that the similarity found in this report between stages of AKIN and KDIGO may be due to the predominance of patients lately referred to a nephrologist with very high levels of SCr. Zeng et al. [34] believe that the KDIGO definition has the highest estimated incidence of AKI, due to more frequent identification of patients with stage 1 AKI and the predominance of available patients with low baseline SCr in which AKI may be defined by a 50% increase over baseline. Zeng et al. [34] also showed that in AKI patients, the lower the level of baseline estimated GFR, the higher the incidence of stage 3 was and the lower the incidence of stage 2 of KDIGO criteria.

Regarding NC, we separated in two groups of 56 and 47 patients, using a cut-off of 4 days (≤ 4 days and > 4 days, respectively), considering median time of 4 [2–7] days. The groups were very similar. SCr was 4.10 mg/dL in the delayed NC group compared with 3.10 mg/dL in the group with NC ≤ 4 days. Mortality was 74.46% in the NC > 4 days group, compared to 50% in NC ≤4 days (*p* = 0.011). In the logistic regression model, delayed NC was associated with death after adjustments [OR 2.66 (95% CI, 1.36–4.35), *p* = 0.001]. Haemodialysis was marginally associated with mortality [OR 1.38 (95% CI, 1.01–2.53), *p* = 0.047], as was SCr [1.30 (95% CI, 1.02–1.71), *p* = 0.039]. A higher maximum SCr was found in the non-survivor group. It has been known since the AKIN report that even minor changes in SCr are associated with increased mortality [11]. The importance of early referral to nephrologist was first described by Metha et al. [24] and then by many other researchers, usually evaluating ICU AKI patients. Metha et al. [24] reported that delayed NC (≥ 2 days) was associated with increased mortality in critically ill AKI patients but this effect was not sustained after propensity score adjustments. They showed that patients with delayed NC ≥ 2 days and ≥ 4 days were associated with mortality [OR 2.5 (95% CI, 1.1–5.9) and 3.2 (95% CI, 1.1–9.4), respectively]. Meier et al. [19] assessing noncritically ill patients observed similar results using a reference of 5 days and reported that, after adjustments, mortality was associated with longer time to nephrologist referral [OR 1.8 (95% CI, 1.36–2.48)] when patients were referred between 6 and 10 days. Balasubramanian et al. [18] reported that early NC was associated with reduced risk of further decrease in kidney function, but they did not find an association of early NC and mortality. However, in that study in the intervention group a NC came too soon, at a median time of 13 h after AKI diagnosis and levels of SCr were slight elevated at NC 1.7 and 1.8 mg/dL in the intervention and control groups, respectively. Ponce et al. [9] in an observational, prospective study observed that NC was associated with increased mortality after adjustment in a multivariable analysis [OR 1.32 (95% CI, 1.16–2.9)]. Costa e Silva et al. [10] conducted an observational, prospective study assessing 366 critically ill AKI patients and observed a higher mortality in delayed NC (91.0, 71.9, and 55.3% in the delayed NC, early NC, and no NC groups, respectively), even after propensity score adjustments.

There are some limitations to our study, such as its retrospective design, reduced number of patients, and that it was performed in a single centre. It is possible that some patients may have died before the initiation of any nephrology assessment. The primary care team might use different patterns for a nephrologist call based on perception of illness severity, in potential evolution for dialysis, and did not assess for mortality risk in cases of slight elevation of SCr. In the present study, we assessed only AKI patients followed by a nephrologist team, which might be biased towards more complex cases or those with some other organ system dysfunction and influenced by the different clinical practice of some nephrologists in selecting patients. We used imputed or commonly used surrogate estimates of baseline kidney function that can result in substantial misclassification of AKI. Because this descriptive study was not designed to investigate time of referral to a nephrologist in many patients, this data could not be assessed in 149 patients.

Our study also has some strengths. It demonstrates the reality of a routine medical practice in a low-income centre in which referral to a nephrologist is delayed and how this can negatively impact the outcomes of AKI patients. Moreover, it demonstrates the altered performance of the KDIGO criteria under these circumstances. The nephrologist team produced specific chart protocols from all patients followed in the study period that allow data assessment even in retrospective study designs, thus minimizing missing laboratory and clinical data.

## Conclusion

In noncritically ill AKI patients in a low-income centre delayed NC occurred in the majority of patients assessed (68.93%) and was associated with mortality even after adjustments. AKIN and KDIGO performed similarly, implying that high SCr baselines could interfere with AKI criteria. Haemodialysis was also marginally associated with mortality. It is not possible to verify influence of delayed NC in mortality given the study limitations, selection bias, and other factors, such as severe AKI cases and residual confounding effects.

Further prospective randomized studies might support effect of timely NC in renal outcomes associated with AKI. A prospective pilot study [18] has not showed better results, probably due to the study design. Conversely, even without robust trials, highlighting the association of timely NC with mortality is imperative when training primary care physicians and other health-care givers in low-middle income countries; this can raise awareness, facilitate sharing of knowledge, and provide practical management of AKI.

## Data Availability

The data that support the findings of this study are available from the corresponding author, GN, upon reasonable request.

## Abbreviations

AKI: Acute kidney injury
AKIN: Acute Kidney Injury Network
BUN: Blood urea nitrogen
CI: Confidence interval
GFR: Glomerular filtration rate
ICU: Intensive care units
IQR: Interquartile ranges
KDIGO: Kidney Disease: Improving Global Outcomes
LMIC: Low-middle-income countries
MDRD: Modification of Diet in Renal Disease
NC: Nephrologist consultation
OR: Odds ratio
RIFLE: Risk, Injury, Failure, Loss, and End-Stage Kidney Disease
SCr: Serum creatinine
SD: Standard deviation
SPSS: Statistical Package for the Social Sciences
